# Community correlates of change: A mixed-effects assessment of shooting dynamics during COVID-19

**DOI:** 10.1371/journal.pone.0263777

**Published:** 2022-02-23

**Authors:** Nicole J. Johnson, Caterina G. Roman

**Affiliations:** Department of Criminal Justice, Temple University, Philadelphia, Pennsylvania, United States of America; Peking University Shenzhen Graduate School, CHINA

## Abstract

This study examines changes in gun violence at the census tract level in Philadelphia, PA before and after the onset of the COVID-19 pandemic. Piecewise generalized linear mixed effects models are used to test the relative impacts of social-structural and demographic factors, police activity, the presence of and proximity to drug markets, and physical incivilities on shooting changes between 2017 and June, 2021. Model results revealed that neighborhood structural characteristics like concentrated disadvantage and racial makeup, as well as proximity to drug markets and police activity were associated with higher shooting rates. Neighborhood drug market activity and police activity significantly predicted changes in shooting rates over time after the onset of COVID-19. This work demonstrates the importance of understanding whether there are unique factors that impact the susceptibility to exogenous shocks like the COVID-19 pandemic. The increasing risk of being in a neighborhood with an active drug market during the pandemic suggests efforts related to disrupting drug organizations, or otherwise curbing violence stemming from drug markets, may go a long way towards quelling citywide increases in gun violence.

## Introduction

Declared a global pandemic by the World Health Organization in March 2020 [1], COVID-19 has had wide-reaching effects on many aspects of daily life for people around the globe. Not only has the virus itself sickened or killed millions, the mitigation strategies enacted by governments [2], from the local level upward, have disrupted governmental, economic, and other institutional functioning, and altered social and behavioral dynamics at an unprecedented scale. Crime is among the social phenomena thought to be impacted by the pandemic [3]. Since the early days of the pandemic, criminologists have sought to describe and explain the patterning of crime in the wake of COVID-19, particularly as a response to mitigation strategies such as stay-at-home orders [4–6]. These efforts have been fruitful in discovering broad city-level trends that persist across space, and in identifying those that do not. However, the emphasis on macro-level (in this context, city-scale) change does not allow for an understanding of why neighborhoods *within* a city might differ in crime change during this global pandemic [7]. Furthermore, despite many studies finding evidence of a crime drop in certain offenses, macro-level research to date has found “paradoxical” trends in gun violence in many jurisdictions [8] that have yet to be explored at the meso-level (sub-city scale).

Focusing on gun violence changes at the census tract level in Philadelphia, the current study adds to the small but emerging body of research that has examined meso-level dynamics of crime during the pandemic [7]. This work is framed primarily by social disorganization theory [9], with additional measures informed by work on incivilities and crime [10], routine activities theory [11] and environmental criminology [12], and seeks to understand not only the meso-level variation in shooting trends during the pandemic, but also the relevant community features that predict this variation. Meso-level research on gun violence during COVID-19 is critical for local policy development, but also for our understanding of whether traditionally robust social disorganization predictors are similarly robust during a global pandemic. Furthermore, testing the relative impact of various theoretically-informed measures will aid in our understanding of the community features most predictive of shooting changes during COVID-19. The paper is organized as follows. First, the background section describes research to date on the link between COVID-19 and violent crime, as well as the theoretical framework of the current study. Next, the methodological approach is described, in addition to the results of the analytical models. Finally, the implications of the results are discussed, followed by concluding remarks.

## Background

### Relevant studies of COVID-19 and violent crime

The relatively short period since the onset of the COVID-19 pandemic has seen a multitude of COVID-19-related studies as scholars rush to understand the relationship between the pandemic, specifically its mitigation policies and crime. These studies have examined multiple crime types across different jurisdictions in the United States [4–7, 13], Australia [14], Central America [15], and the United Kingdom [16] using a variety of methodologies.

Studies exploring the potential impacts of the pandemic on violent crime have suggested mixed results. For instance, two studies using police-recorded crime data for a sample of large cities found diverging results. Using Seasonal Autoregressive Integrated Moving Average (SARIMA) models of several crime types in 16 U.S. cities between 2016 and January 20, 2020, Ashby [5] found there was no evidence of significant changes in serious assaults (either street assaults or those occurring in the home) based on comparison of the expected and observed frequency of assaults. However, he noted heterogeneity between cities in the effect of COVID-19 on crime. A similar study conducted a time series analysis of 25 large U.S. cities, finding on average that most experienced an immediate reduction in crime incidents after the pandemic began [6]. Importantly, the author found that Philadelphia was one of 6 cities examined that experienced at least a 35% decline in overall crime. However, dynamics varied by crime type. He found that while robbery, aggravated assault, and simple assault declined post-pandemic, homicides and shootings did not. Abrams [6] posited that homicides and shootings would likely follow different trends post-COVID-19, specifically noting the contribution of gangs and drug-markets to violent crime rates.

The impact of stay-at-home orders appeared to have a differential impact on *types* of shootings as well. Kim and Phillips [17] used time series analysis to examine weekly changes in fatal shootings, nonfatal shootings, nonfatal shootings with and without injury, and gang- and non-gang related shootings from January 2017 through October 5, 2020 in Buffalo, NY. Results from ARIMA models indicated that both fatal and nonfatal shootings increased after the implementation of stay-at-home orders, though the effect on fatal shootings was abrupt and temporary compared to nonfatal shootings and gang-related shootings, which sustained prolonged increases.

Scholars have also used ARIMA models to estimate the impact of social distancing orders in Los Angeles on gang crime specifically [18]. Analyzing weekly reported crime data from January 1, 2016 through September 28, 2020, the authors found no significant change in the amount of violent crime, robbery or assault, or gun violence attributed to gangs after March 20, 2020 (the first day of shelter-in-place orders). In addition, they found no evidence of changes in the spatial distribution of gang-related crimes following the onset of the stay-at-home orders.

A report examining the effect of COVID-19 on homicide found evidence of a decrease in homicides immediately following many cities’ lockdowns [19]. Examining change in homicides between January and June 2020 for 64 US cities, the authors found that the average monthly homicide rate grew by 6% in 2020, as compared to each city’s 2017–2019 average for the same period. However, when examining month-to-month variation, they found that the homicide rate in most cities, including Philadelphia, increased during January, February, and March, yet decreased in April and May after the lockdowns. Despite this finding, the authors foreshadowed a trend reversal for the remainder of 2020, citing relaxation of social distancing policies, public resource exhaustion, tightening municipal budgets, the end of unemployment relief, and social unrest resulting from the murder of George Floyd as potential reasons for an uptick. This latter event was included in another report that aimed to understand the impact of not only the pandemic, but the widespread social unrest brought on by the killing of George Floyd on May 25, 2020 [20]. Researchers explored weekly changes in 11 different crimes across 27 large cities for January 2017 through June 2020. They converted each crime to a weekly rate per 100,000 city residents, and estimated structural breaks (a statistically significant change) in their time series, adjusting for seasonal effects. For homicide in general, the authors confirmed predictions made in [19]. Their model estimated a structural break around the end of May 2020, when the average weekly homicide rate for the 20 cities in their analysis subsequently increased by 37% through June 2020. Importantly, they noted that Philadelphia, Chicago, and Milwaukee were driving this increase. Most relevant to this study, they analyzed the weekly gun assault rate for 17 cities, finding an estimated structural break around March 2019, one year prior to the onset of COVID-19. On average, they found that while gun assaults rose during the early summer of 2020, this increase was not significantly different than the prior year.

All studies discussed thus far have been situated at the city level. We are aware of only one study that seeks to understand the pandemic-crime relationship at a lower level of aggregation [7]. Campedelli and colleagues’ [7] work was motivated by the understanding that crime is clustered based on certain features of the social and ecological environment [21]. They explored the impact of COVID-19 on daily trends of burglary, robbery, assault, and narcotics rates between January 1, 2018 through May 17, 2020 across 77 Chicago community areas. Their analysis comprised two parts. First, they used Structural Bayesian Time Series models to predict significant differences in each crime type during the post-COVID intervention period. Next, they used Firth’s logistic regression to predict the estimated reductions in each crime type with four sets of covariates: crime-related variables, socioeconomic variables, health and demographic variables, and variables testing a joint-reduction of different crime types. With respect to assault, they found that the poverty rate did not significantly predict assault rate reductions, yet their measure of income inequality did. Additionally, they found that the likelihood of a community experiencing a significant reduction in assaults was also associated with a significant reduction in narcotics offenses, and vice-versa, suggesting there may be a similar process linking the two crime types [7].

The work exploring the impact of the pandemic on crime is surely in its infancy. However, two tentative conclusions can be drawn from prior work that motivate the current study. Those are that the rates of gun violence appeared to remain stable or increased in many jurisdictions after the onset of the pandemic [8, 17, 20], and that the relationship between the pandemic, its policies and crime was not experienced in a similar manner across places [5]. Following [7], the current study thus tests whether these findings are true of Philadelphia neighborhoods as well, and goes a step further in testing the relative predictive power of groups of theoretically-relevant community correlates on shooting rates over time.

### COVID-19 and gun violence in Philadelphia

In 2020, Philadelphia was battling multiple public health crises at once. A week after the first COVID-19 case was announced in Philadelphia [22], courts closed and law enforcement temporarily restrained from making low-level arrests [23], in order to curb the spread of the virus. By March 23, Governor Tom Wolf issued a stay-at-home order to multiple counties in Pennsylvania, including Philadelphia [24]. As of this writing, more than 180,000 cases and 4,000 deaths related to COVID-19 have been reported in Philadelphia by the New York Times [25]. At the same time when the pandemic was in full swing, some hospital trauma centers were seeing a larger share of gun violence victims than before the stay-at-home orders were enacted [26]. Researchers conducted a study in Philadelphia using ARIMA time series models to determine if there was a significant change in shootings following the enactment of COVID-related containment policies and protests for racial justice [27]. They found that weekly rates of shooting victims nearly doubled after the March 23 lockdown compared to weekly rates in prior years [27]. By year-end 2020, more than 2,240 people were victims of gun violence in Philadelphia [28].

The primary focus of this study is on the time period that we describe as spanning the COVID-19 pandemic. However, events in addition to the pandemic occurred within this time frame. Similar to other U.S. cities, George Floyd’s murder in late May brought days of active protests across Philadelphia, in addition to changes in police activity. How these events played out in the city may correspond to the finding that Philadelphia was one of three cities driving a large increase in average weekly homicide rate after the end of May, 2020 [20]. Five months after the death of George Floyd, Walter Wallace was shot by police officers in West Philadelphia, spurring another round of protest activity in the city that resulted in numerous injuries and arrests [29].

### Theoretical framework

This article incorporates multiple predictors of community violent crime rates to assess their relevance in explaining neighborhood differences in shooting changes over time. Social disorganization theory [9] influences several key measures tested in this analysis, as many of the theory’s constructs have proven robust to empirical testing over time [30]. Social disorganization theory was developed in the early twentieth century as an ecological explanation for why some communities in Chicago had higher delinquency rates than others, and why these spatial patterns persisted over time [31]. According to the theory, neighborhood structural characteristics, such as economic deprivation, residential turnover and ethnic heterogeneity, impede the capacity of neighborhoods to regulate themselves through informal social control [32]. Since its inception, the main tenets of social disorganization theory have been extended by scholars who have posited mechanisms like weakened social ties [33], or depressed collective efficacy [32, 34] as processes linking neighborhood structure to higher crime rates.

While the original formulation of social disorganization theory posits ethnoracial heterogeneity as a key disorganizing factor at the neighborhood level, research on urban crime has also demonstrated that racially segregated neighborhoods, particularly majority Black neighborhoods, may incur higher rates of violent crime as a result of city-wide stratification of other neighborhood conditions, such as poverty [35–37]. This stratification of poor neighborhood conditions may link to higher violent crime in minority neighborhoods as a result of cultural adaptations, or social isolation preventing effective community control [36, 38, 39]. There is evidence in Philadelphia of racial disparities in gun violence victimization, even in neighborhoods without crippling levels of disadvantage [40]. Researchers [40] found that in the highest income Philadelphia census blocks, Black residents had a relative risk of firearm assault nearly 16 times greater than that of white residents, suggesting there is another mechanism leading to disparities in victimization above and beyond structural disadvantage [40].

There has been general support for social disorganization theory, and its extensions, as an explanation of community crime rates, and violent crime in particular [30, 33, 41]. However, certain indicators have received more support than others. For instance, Magee [42] analyzed the effects of census tract-level measures of disorganization in 2014 on street-level shooting incidents in 2015 in Indianapolis. She found a positive relationship between tract-level concentrated disadvantage and percent Black and shootings, controlling for street-level disorder and collective efficacy. She did not observe significant relationships between tract-level residential mobility or ethnic heterogeneity, however. Similar to the current study, Kubrin and Herting [43] analyzed the effects of social disorganization indicators on neighborhood homicide *changes* over time. Estimating growth curve models of yearly homicides between 1980 and 1994 in St. Louis, they found that the neighborhood level of disadvantage was significantly related to initial levels of three types of homicide (general altercation, felony, and domestic killings), and was related to changes in general altercation and felony killings. Furthermore, residential stability was significantly related to initial levels of felony and domestic killings only, and did not predict trends in any of the three homicide types examined. The current study draws on both Kubrin and Herting’s [43] analysis and other neighborhood effects research to assess whether neighborhood-level shootings followed different processes before and after the onset of COVID-19, and if so, which social disorganization measures best predicted these changes.

Relevant to the current study, the presence of drug markets has been tested as a mediator of neighborhood structure on violent crime rates at the neighborhood level [44]. Analyzing 74 Miami census tracts, Martinez, Rosenfeld and Mares [44] found that social disorganization dimensions of neighborhoods (ethnic heterogeneity, residential instability, and concentrated deprivation) had a direct effect on neighborhood levels of aggravated assault, in addition to an indirect effect on assault through their measure of drug markets. The general pattern of findings remained whether they used drug arrests or drug overdoses to measure drug markets, suggesting that there was something about drug markets that may themselves be disorganizing, further inhibiting social control, and amplifying violent crime. This finding is also consistent with the notion in environmental criminology of “crime attractors”, in the sense that people are drawn specifically to areas with drug markets to engage in illicit activity [12]. According to this perspective, drug markets may serve as an attractor for elevated crime rates as they attract people to buy and sell drugs, which may produce scenarios that increase the levels of violence in those places. Some research has even found that drug markets attracting buyers from further distances have significantly more violence than markets with local buyers and sellers, owing to the interactions between unfamiliar buyers and sellers [45]. In addition to the draw of criminogenic activity to drug market locations, routine activities theory would suggest that drug market environments may be associated with higher crime rates as they may host fewer capable guardians on the street [11, 46]. The conditions that make drug markets more likely to exist in the first place, such as a lack of place managers at local businesses who are able to regulate behavior, could also translate to more opportunities for violence in these settings. Research on the drug market-violence link has indeed found that drug markets produce situations in which drug dealers and users are vulnerable to predation without recourse to formal authorities, making them more likely to engage in self-help behavior [47; see also 48, 49]. Furthermore, expanding drug markets connected to the opioid epidemic have been suspected to play a part in recent spikes in homicide as well [20, 50].

In addition to metrics of neighborhood disorganization and the criminogenic nature of drug markets, measures capturing the state of the physical environment have been found to influence neighborhood crime rates. Physical incivilities, or signs of disorder (e.g. graffiti, abandoned vehicles, street litter, vacant houses), are thought to relate to higher crime via the signals they send to potential guardians and offenders regarding neighborhood control [51, 52]. That is, incivilities may serve as visual markers of the absence of social control in neighborhoods, and may translate to lower willingness among residents to engage in informal social control [10]. Physical incivilities may also cue would-be offenders that neighborhood residents lack the ability to regulate the community, thus emboldening them to action. Some research has shown that neighborhood incivilities link to higher crime [10, 53, 54]. For instance, Sampson and Raudenbush [54] found that tract-level incivilities (measured via systematic social observation) significantly predicted later homicide and robbery in Chicago. Taylor [10] similarly found that incivilities in Baltimore neighborhoods significantly predicted homicide levels. Examining gun violence specifically, Magee [42] found that physical disorder (measured as the number of abandoned homes) predicted higher rates of shootings, though at a smaller unit of analysis (street segment). In addition to measures of neighborhood status and composition, the current study attempts to answer whether more physical blight in Philadelphia neighborhoods predicts higher levels of gun violence before and after the onset of the pandemic.

The current study seeks to answer five related research questions:

1. *What was the typical change in shootings before and after the onset of the COVID-19 pandemic and how do changes in the two periods compare to each other*? Based on prior research on gun violence and COVID-19, we expect that the average rate of change in the post-COVID period will be steeper than that of the pre-COVID period.
2. *Did neighborhoods differ in shootings at the onset of COVID-19*? Literature on the concentration of crime at places has demonstrated that crime is not uniformly distributed across a city, but rather tightly bound to certain types of places [21]. Therefore, we expect that there will be neighborhood-differences in the rate of shootings at the beginning of the pandemic.
3. *If there is between neighborhood variability in shootings at the start of the pandemic*, *what community-level correlates best predict this variability*? Drawing from the literature discussed above, we expect that communities that are characterized by more social disorganization and physical incivilities, high drug arrest rates, and that are spatially proximate to drug markets and high-shooting, high-disadvantage communities will exhibit higher rates of gun violence.
4. *Did neighborhoods differ in their rate of change in shootings before and after the COVID-19 pandemic began*? Similar to the rationale for neighborhood differences at the start of COVID-19, we also expect that neighborhoods will differ in their rate of change over the course of the pandemic.
5. *If there is between neighborhood variability in rate of change*, *what community-level correlates best predict this variability*? Some scholars have advanced theories of crime change during the pandemic, including drug market and gang-related causes, in addition to public disorder resulting from protest activity over racial equality (e.g. [20]). Based on the existing literature, we expect that the effects of neighborhood structural disadvantage and composition, drug market activity and proximity, and physical incivilities on shooting rates will change after the onset of COVID-19.

## Methods

### Study area and unit of analysis

The site of this study is Philadelphia, PA, a city in the northeastern United States with a population of roughly 1.5 million people [55]. The city is racially and ethnically diverse, with a nearly equal percentage of Black and white residents (42.1% and 40.7%, respectively) and significant Hispanic or Latino population (14.7%). An estimated quarter of Philadelphia residents speak a language other than English at home. Philadelphia often ranks higher than the national average on measures of poverty. According to the 2019 American Community Survey 5-year estimates, the median household income in Philadelphia was almost $46,000, compared to $68,700 nationwide. Similarly, the percent of Philadelphians in poverty was estimated at more than 24%, compared to 10.5% in the country as a whole.

As the focus of this research is on neighborhood-level change, census tracts were used as the spatial unit of analysis to approximate Philadelphia neighborhoods. Census tracts are often used in neighborhood-level research because they are generally stable over time, and because they facilitate the aggregation of census data to each unit with ease. This study uses the 2010 Philadelphia census tract boundaries, which were obtained via the Open Data Philly data portal (see S1 Table for data sources). Out of the original 384 census tracts, seven were removed from the analysis due to having a population of zero, according to the 2014–2018 American Community Survey 5-year estimates. A further four tracts were removed from the analysis because they were comprised entirely of major parks, leaving a final sample size of 373 census tracts. The average size of a Philadelphia census tract is roughly 0.35 square miles, with an average residential population of 4,220 people. Because shootings are not a frequent occurrence, even in the highest crime neighborhoods, the detection of weekly or even monthly changes in shootings is very difficult. Therefore, the temporal units used in the study are bimonthly units. The study time period extends from January 2017 through June 2021, resulting in 27 time points. As described more fully in the analytic approach section, the final analytical dataset nested the 27 time points within 373 census tracts, resulting in 10,071 observations.

### Data and measures

#### Dependent variable

The outcome of interest in the study is the number of shootings occurring per bimonthly period, where shootings refer to shooting victims. Shooting data for Philadelphia were obtained via the Open Data Philly data portal. These data are publicly released by the Philadelphia Police Department daily, and contain both criminal and officer-involved shootings. For this research, shooting victim data were downloaded as a shapefile and imported into ArcGIS Pro 2.6.0 for aggregation to the Philadelphia census tracts. After the removal of officer-involved shootings, the shapefile included a total of 8,122 shootings occurring between January 1, 2017 and June 30, 2021. 96% of shootings were geocoded to a Philadelphia street block or intersection and joined with a Philadelphia census tract, exceeding minimum acceptable thresholds [56, 57]. Once each shooting was connected to a census tract, the resulting data file was imported to R, where it was collapsed into total counts of shootings per tract, per bimonthly period.

#### Independent variables

*Structural predictors*. Measures were included in the analyses that aimed to capture the role that neighborhood structure plays in differences in the evolution of shootings over time. Indicators of social disorganization have taken many forms in communities and crime research. This study included measures of concentrated disadvantage, residential instability and an index reflecting the foreign-born population to capture measures of social disorganization [32, 33, 41]. Concentrated disadvantage was measured as the average of four z-scored census items: the percent of the population in poverty, in female headed households, unemployed, and the percent of households receiving public assistance (Cronbach’s α = 0.85). These items, along with all other variables used to construct the structural indicators, were obtained from the 2014–2018 American Community Survey 5-year estimates. Residential instability was measured as the percentage of renter-occupied households within the census tract. The foreign-born index was created as the average of the z-scores of the percent foreign born population and percent of the population reporting that they spoke a language at home other than English (Cronbach’s α = 0.84). An ethno-racial heterogeneity index was also created for inclusion into the statistical models, yet this variable correlated highly with the majority Black variable described below, and was thus excluded from all models.

Finally, a variable measuring whether a neighborhood was more than 70% Black (*PK Black)* was included as a dichotomous predictor of shootings when COVID-19 began and its change over time. Peterson and Krivo [35] used 70% as a threshold for defining the ethnoracial makeup of census tracts. This variable was included as a theoretical predictor that reflected the residual risk of gun violence victimization above and beyond economic disadvantage that has been documented in prior research [e.g. 37, 40]. To further aid the interpretation of model coefficients, the concentrated disadvantage, foreign-born, and instability indices were z-scored prior to inclusion in the models.

*Drug markets*. Supply- and demand-side pressures for illicit narcotics could have theoretically influenced the interactions among drug trade organizations and between sellers and buyers, potentially altering the landscape for violence in neighborhoods with significant drug activity. However, it is difficult to know for certain what effect COVID-19 has had on drug markets [58]. To test whether high drug market activity predicted changes in shooting rates after the pandemic began, an indicator of whether a neighborhood experienced a high rate of drug arrests (drug sales and possession) between 2017 and the end of 2019 was included in the models. Due to shifts in arrest policy at the Philadelphia Police Department, drug arrests made in 2020 were not included in the determination of tracts with high drug market activity. Drug arrest counts collapsed to the census tract were provided by the Philadelphia District Attorney’s Office. The drug market measure was dichotomized due to extreme skew (skewness = 8.99), as drug activity is highly concentrated. The measure was created by first rating the pooled number of drug arrests for 2017–2019 per 1,000 population for each census tract, then assigning the tracts falling above the 90^th^ percentile of this distribution a value of one, and to the rest a value of zero. Drug arrests have been used to estimate drug markets in prior work [59–61]. The tracts identified as having high-drug activity through this process corresponded closely with previously identified drug markets in Philadelphia [62]. Furthermore, erring on the side of choosing a higher percentile (rather than lower) ensures that the measure is capturing those spaces that have very active drug markets.

*Distance to drug markets*. As stated previously, drug markets are an example of a “crime attractor” in environmental criminology, whereby the area serves as an attractor point for illicit activity, as users and sellers go about their routine activities [12, 63]. It was expected that neighborhoods with active drug markets would have relatively higher shooting rates than those without, and likewise, that neighborhoods located closer to drug markets would exhibit more gun violence than those situated further away. Neighborhoods in closer proximity to drug markets may experience more shootings as individuals affiliated with the illicit drug trade traverse neighborhood boundaries during their routine activities, or if norms regarding violence and gun carrying spillover to nearby areas [64]. To create a distance to drug markets measure, the Euclidean distance in miles was calculated between the centroid of each census tract in Philadelphia to the centroid of each of the drug market tracts identified in the previous section. The total distance in miles was then summed for each tract, reflecting a total distance from all identified drug markets. Tracts with larger distances were located further away from all drug markets, while those with smaller distances were located closer to all drug markets. The measure was z-scored prior to inclusion in the models.

*Physical incivilities*. As noted above, physical incivilities have previously been linked to crime at various levels of aggregation, such as the census tract and census block levels [52, 54, 65]. The current analysis tests the relative importance of blighted physical environment on shootings and shooting changes following the onset of COVID-19. There have been many studies in the environmental criminology literature that have directly measured aspects of the built environment and their relationship with crime [61, 66]. 311 calls have also been previously used in research to measure physical disorder [67, 68]. The current study uses 311 calls for service to capture a community-wide measure of physical incivilities, as they corresponded with other observed measures of physical blight in Philadelphia (i.e. % vacant housing units and observed street litter), and because they reflected perceptions of issues within neighborhoods, most likely from the viewpoint of residents themselves [67].

To construct the neighborhood measure of physical incivilities, calls were obtained via the Open Data Philly portal, and geocoded to Philadelphia census tracts. Five public space call types were included in the measure: those regarding graffiti removal, abandoned vehicles, vacant lot clean up, vacant house calls, and streetlight outages, as these were identified as the most frequent quality-of-life call types in 2019 by the Philly 311 office in another project. In an effort to standardize the metric, the calls were pooled between 2017 and June 30, 2021, divided by the total population for each census tract and multiplied by 1,000 to reflect a rate of calls per 1,000 residential population. This measure was then z-scored before inclusion in the models.

*Police activity*. A time-varying measure of the number of police investigations occurring in each tract, in each period, was included in the models to capture changes in police activity that occurred during the pandemic. This measure of “police investigations” consists of police car stops and pedestrian stops. Including this variable also serves to mitigate the endogeneity bias associated with using police-driven measures of drug activity [44]. At the city-level, police stops have been found to vary over time with measures of routine activities of citizens, as estimated by major events or days of the week [69]. As noted in the beginning of this article, the early days of the pandemic saw a curtailing of police activity in order to prevent the spread of the virus. Additional analyses (not shown) also revealed that police stops decreased across the city after the death of George Floyd in late May, 2020, though they steadily rebounded through the end of the year. Variations in police activity across neighborhoods might predict corresponding variations in shooting rates, as a less visible police presence could potentially alter the cost/benefit analysis of potential shooters. Police investigations data were obtained via the Open Data Philly portal. Because investigatory stops can often include people who do not live within the area where they are stopped, the measure was not rated by the residential population of the census tracts. The stop counts were z-scored prior to inclusion in the models.

*Additional controls*. Spatially-and temporally-lagged variables were included to control for the confounding influence of surrounding tracts and previous time points, respectively. A global Moran’s I test was performed on the dependent variable, revealing significant positive spatial autocorrelation (I = 0.67, pseudo-p < .001). This is consistent with past research revealing that neighborhood levels of violent crime are linked with that of nearby neighborhoods [70]. Some scholars have posited that violence, particularly gun violence, diffuses across neighborhood boundaries through a process of social contagion [71]. A spatially-lagged dependent variable was therefore included in the model to capture the potential influence of surrounding tracts on each focal tract. This variable was created in Geoda 1.14.0, using a first-order Queen’s contiguity spatial weights matrix, and translates to the average number of shootings occurring in the tracts immediately surrounding a focal tract in each bimonthly period.

To account for the potential influence of the number of shootings in the preceding time period, a first-order temporally-lagged shooting variable was also included in the models [72]. Because the inclusion of a temporally lagged shooting variable creates a missing value at the first time point, only 26 time points are included in the models with this predictor, resulting in a corresponding drop in the number of observations. Past research has revealed that disadvantaged neighborhoods are not unaffected by their context, but rather experience the compounding effects of being surrounded by other disadvantaged neighborhoods [73, 74]. These “deserts of disadvantage” are poorly situated in that the relative isolation and dearth of resources can drain community capacity to regulate itself through informal social control [74]. This study thus accounts for the deleterious influence of a neighborhood’s own structural disadvantage, as well as the added impact of disadvantage in its immediate environment, by the inclusion of a spatially-lagged measure of concentrated disadvantage. This variable was also created in Geoda 1.14.0 using the same spatial weights matrix described above. Both spatially lagged predictors were z-scored prior to inclusion in the models. Table 1 details the descriptive statistics of the variables used in this analysis.

**Table 1:** Descriptive statistics.

Variables	Description	N	Mean	SD	Min	Max
*Level one*						
Shootings	Count of shootings per tract per bimonthly period	10,071	.707	1.42	0	17
Timepre	Pre-COVID linear time slope	10,071	-7.04	6.48	-19	0
Timepost	Post-COVID linear time slope	10,071	1.04	2.03	0	7
Timepost^2^	Post-COVID quadratic time slope	10,071	5.19	12.10	0	49
Police stops	z-scored count of police investigatory stops in each tract per bimonthly period	10,071	0	1	-.69	9.78
Temporal lag shootings	T-1 lag of shootings	9,698	.69	1.40	0	17
Spatial lag shootings	z-scored average shooting count in adjacent tracts	10,071	0	1	-.81	9.14
*Level two*						
Concentrated disadvantage	z-scored average of four z-scored census items: % population in poverty, in female headed households, unemployed, households receiving public assistance	370	0	1	-1.65	3.78
% Renters	Percent renter-occupied households	372	0	1	-2.26	2.75
Foreign born	z-scored average of % foreign born and population speak language other than English	373	0	1	-1.22	4.24
PK Black	Peterson-Krivo >70% Black indicator	373	.28	.45	0	1
Drug market	>90^th^ percentile drug arrest rate for 2017–2019	373	.10	.30	0	1
311 calls	z-scored pooled 311 call rate, 2017-June 2021	373	0	1	-1.04	16.84
Spatial lag Concdis	z-scored average concentrated disadvantage score in adjacent tracts	373	0	1	-1.90	2.62
Distance to drug markets	Total distance in miles between each tract and all drug market tracts	373	0	1	-1.24	3.56
Total population	Total residential population	373	4220.61	1687.59	409	9034

Note. N refers to the number of observations at level one and tracts at level two. SD refers to standard deviation. Concdis refers to concentrated disadvantage.

### Analytic strategy

A key goal of this research is to understand the inter-neighborhood differences in intra-neighborhood change in shooting rates before and after the COVID-19 onset in Philadelphia. This required an analytical approach that would take into account the correlated errors within tracts over time. One can account for clustered errors by incorporating an extra layer of nesting to the analysis via a hierarchical linear model (HLM), also called a mixed effects model [75, 76]. A mixed effects model was deemed the appropriate model choice for this study, because the interest was to estimate the underlying pathway of shootings before and after the onset of the pandemic, while allowing for an exploration of variability among the individual pathways of each neighborhood. Past research has used this analytical approach to characterize shooting or homicide trajectories over time [43, 72]. The count distribution of the outcome variable further required the specification of a generalized linear model. Therefore, mixed effects negative binomial regression models, nesting time within neighborhoods, and accounting for the count distribution of the outcome variable, were specified for the current analyses [76]. This model was appropriate for the current study, as the variance of the outcome was greater than the mean (mean = 0.7, variance = 2.0). Furthermore, a mixed effects Poisson model was run along with the negative binomial model, with the latter resulting in a better fit.

The entire time period under study spans 27 time points, from January/February 2017 through May/June 2021. In the mixed effects modelling framework, the functional form of the relationship between the phenomenon of interest and time is coded in the time variable itself [77]. For example, a linear increase in shootings over time would be characterized by a time variable with equally spaced units spanning the period of analysis. Incorporating higher-order polynomial terms for time (e.g. time^2^ or time^3^) allows one to capture departures from a constant rate of change, or bend(s) in the curve. For the current study, a two-rate model was specified, where separate linear time slopes were used to characterize the pre-COVID (*timepre*) and post-COVID-onset (*timepost*) periods. This meant that the pre-COVID time period was characterized by a linear trend over 20 time points, and the post-COVID onset time period was characterized by a linear trend over 7 time points. The intercept (time 0), was set to March/April 2020 for both time slopes. By coding the intercept in such a way, the main effects in the subsequent models can be interpreted as predicting the shooting rate at the beginning of and immediately following the onset of COVID-19. Using a two-rate, or piecewise approach has been used in other studies examining a range of developmental phenomena [e.g. 78, 79], including trends in disruptive behavior among youth before and after a school shooting [80]. A two-rate model was run for two reasons: first, there was reason to believe, based on prior research, that shootings may have unfurled as a result of a different underlying process during COVID-19 than before. Secondly, the two-rate model was compared with both a model characterized by a single linear time slope, and a model characterized by single linear and quadratic time slope, with the two-rate model resulting in better fit in both instances. Further inspection of the observed shooting trends post-COVID suggested that while shootings have remained high in 2021 relative to prior years, they may be trending downward from the sharp increases in 2020. Therefore, a quadratic *timepost* slope was also included in the models to account for any departures from a linear change post-COVID.

Model specification proceeded in stages corresponding to each research question. First, an empty model (with no predictors) was run in order to decompose the variance in shootings attributed to the level-1 (time-level), and level-2 (neighborhood-level) differences. Specifically, the intraclass correlation coefficient (ICC) was calculated following [81, pp. 122–124], and simply refers to the proportion of total variance in the outcome due to the estimated between-group variance. Next, the unconditional growth model (Model One) was run including only the two linear slopes and one quadratic slope representing the periods before (*timepre*) and after (*timepost*, *timepost*^*2*^) the onset of the COVID-19 pandemic. The unconditional model allows neighborhoods to vary in their intercepts; that is, their level of shootings at the onset of the pandemic (March/April 2020). Model Two introduces the structural variables, drug market predictor, distance from drug markets measure, measure of physical incivilities, police activity, and the spatial and temporal lags to attempt to explain any between-neighborhood variability in level of shootings at the onset of the pandemic. Model Three allows for the *timepre* and *timepost* slopes to vary between neighborhoods to determine whether neighborhoods varied in their rate of change in shootings over each time period in question. To answer the core question of this analysis, interaction terms for each group of neighborhood variables and the time slopes were individually entered into the model (Models Four through Six). Finally, Model Seven includes interaction terms for the police stops measure and the time variables to determine whether the effect of police activity on shootings may have changed during the course of the pandemic. All models were run using the menbreg command in Stata v17.0.

## Results

**Fig 1:**
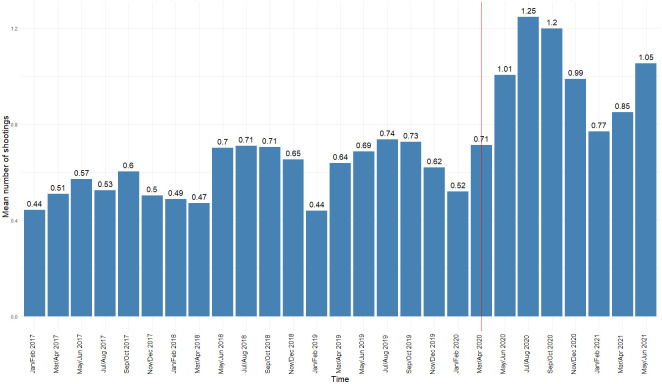
Mean shootings per tract per bimonthly period, 2017-June, 2021. Red vertical line denotes the beginning of the COVID-19 pandemic in March/April, 2020.

Fig 1 details the average number of shootings occurring in Philadelphia census tracts at each time point. The pre-COVID trend displays a subtle increase prior to the onset of the pandemic. However, there is a dramatic and sustained upswing in 2020, with March and April 2020 experiencing 12% more shootings than the same period in 2019. The peak average number of shootings during COVID occurred during July and August 2020, with an average of 1.3 shootings per tract, per bimonthly period. This is nearly 70% higher than the average number of shootings in the same period of 2019. Results from the inferential models are discussed below in context of the relevant research question.

### Research question 1—Comparing change pre- and post-COVID-19

Results from the variance decomposition of the null model revealed that most of the variation in shootings arose from between-neighborhood differences (59.8%), compared to within-neighborhood differences over time (40.1%). Table 2 contains the results from the main effects models. The model containing only the time variables (Model One) revealed that a typical neighborhood had an expected rate of close to zero shootings per person at the beginning of the pandemic, as would be expected given that shootings are a relatively rare event at this spatiotemporal resolution (exp(-9.504) = 0.0001, *p* < 0.001) While both the pre-COVID and post-COVID-onset time slopes were positive and significant, the post-COVID-onset slope coefficient (*b* = 0.195, p < .001) was steeper than the pre-COVID slope (*b* = 0.020, p < .001). Further, the significant negative *timepost*^*2*^ coefficient (*b* = -0.024, p < .001) suggested the post-COVID increase slowed significantly going into 2021. See S1 Fig for a graph of the model-implied average slope. Exponentiating the post-COVID-onset slope coefficient to obtain an incidence rate ratio (IRR) revealed that with each passing period after the start of the pandemic, the rate of shootings in a typical neighborhood increased by a factor of 1.216, or 21.6%.

**Table 2:** Mixed effects models predicting shooting rates in Philadelphia neighborhoods.

Shootings	Model One			Model Two			Model Three		
	*Coef*.	*SE*	*IRR*	*Coef*.	*SE*	*IRR*	*Coef*.	*SE*	*IRR*
Timepre	0.020***	.003	1.02	0.015***	.003	1.02	0.017***	.004	1.02
Timepost	0.195***	.030	1.22	0.221***	.033	1.25	0.228***	.033	1.26
Timepost^2^	-0.024***	.005	0.98	-0.027***	.005	0.97	-0.029***	.005	0.97
Concdis				0.432***	.061	1.54	0.435***	.062	1.55
Renters				0.002	.043	1.00	0.004	.044	1.00
Foreign born				-0.061	.044	0.94	-0.064	.044	0.94
PK Black				0.463***	.094	1.59	0.462***	.095	1.59
Drug market				0.083	.111	1.09	0.090	.112	1.09
Dist. to drug markets				-0.285***	.056	0.75	-0.283***	.057	0.75
Police stops				0.099***	.018	1.10	0.101***	.019	1.11
311 calls				0.040	.023	1.04	0.042	.023	1.04
Temporal lag shoot.				-0.007	.010	0.99	-0.018	.010	0.98
Spatial lag shoot.				0.125***	.019	1.13	0.125***	.019	1.13
Spatial lag concdis				0.449***	.069	1.57	0.454***	.070	1.58
Constant	-9.503***	.092		-9.608***	.058		-9.604***	.058	
*Random effects*									
Variance(cons.)	2.467			0.250			0.246		
Variance(timepre)							0.0003		
Variance(timepost)							0.0029		
N (obs)	10,071			9,620			9,620		
N (groups)	373			370			370		
*LL(df)*	-9346.3(6)			-8756.1(17)			-8750.7(19)		
*AIC*	18704.6			17546.1			17539.3		
*BIC*	18747.9			17668.0			17675.6		

Notes. Concdis refers to concentrated disadvantage. Dist refers to distance.

* *p* < 0.05,

** *p* < 0.01,

*** *p* < 0.001.

### Research question 2—Did neighborhoods differ at COVID-19 onset?

There was significant variation between neighborhoods in their level of shootings during the start of the pandemic, as evidenced by the significant likelihood ratio test comparing the random intercept model with a standard negative binomial regression. This variability among neighborhoods was somewhat unsurprising (but nonetheless important), as years of research in place-based criminology have highlighted the degree to which area context is important and crime concentrates in places [21, 82]. Introducing the community predictors and lag variables explained part of this variation in the intercepts (Model Two). Specifically, there was a 90% decrease in the remaining variance from Model One (variance = 2.47) to Model Two (variance = 0.25).

### Research question 3—What community correlates best predict variability at COVID-19 onset

Of the variables measuring structural characteristics, neither residential instability or foreign-born population were significant predictors of shootings in Philadelphia neighborhoods immediately before the onset of the pandemic. The Peterson-Krivo Black indicator and concentrated disadvantage measure were both significant and positively related to shootings, net of other variables. Because the Census variables were z-scored prior to inclusion in the models, the coefficients are in standard deviation units, meaning, for instance, with every one-standard deviation increase in concentrated disadvantage above the average, shootings were expected to increase by a factor of 1.5 (IRR = 1.54, p < .001).

Contrary to expectations, the drug market indicator did not significantly predict differences in shooting rates at the start of COVID-19. However, the measure of the distance to all drug markets was significant and negative, indicating that with each standard deviation increase in distance over the average, shootings were expected to decrease by roughly 25% (IRR = 0.75, p < .001). The measure of police stops was significantly and positively related to shootings prior to the start of the pandemic. A one-standard deviation unit increase in police stops above the average implied a nearly 10% increase in shootings (IRR = 1.10, p < .001). Neighborhood physical incivilities, as measured by the 311 calls rate, did not significantly predict tract shooting differences.

The effects of the spatial and temporal lags of the dependent variable and of concentrated disadvantage were mixed. The temporal lag of shootings was not significant in this model, indicating that shootings may not have been significantly affected by shootings in the previous bimonthly period. The same could not be said for neighborhood ecological context, however. Both the spatial lag of shootings and of concentrated disadvantage were significantly and positively related to shootings, meaning that a neighborhood’s predicted shooting rate at COVID onset was at least partially explained by the shooting rate and level of disadvantage of its immediate neighbors. The lagged concentrated disadvantage variable is slightly larger than the effect of concentrated disadvantage in the focal neighborhood. A one-standard deviation increase above the average level of concentrated disadvantage in surrounding tracts was associated with an expected 57% increase in shootings in the focal tract (IRR = 1.57, p < .001).

### Research question 4—Did neighborhoods differ in their rate of change pre- and post-COVID-19?

**Fig 2:**
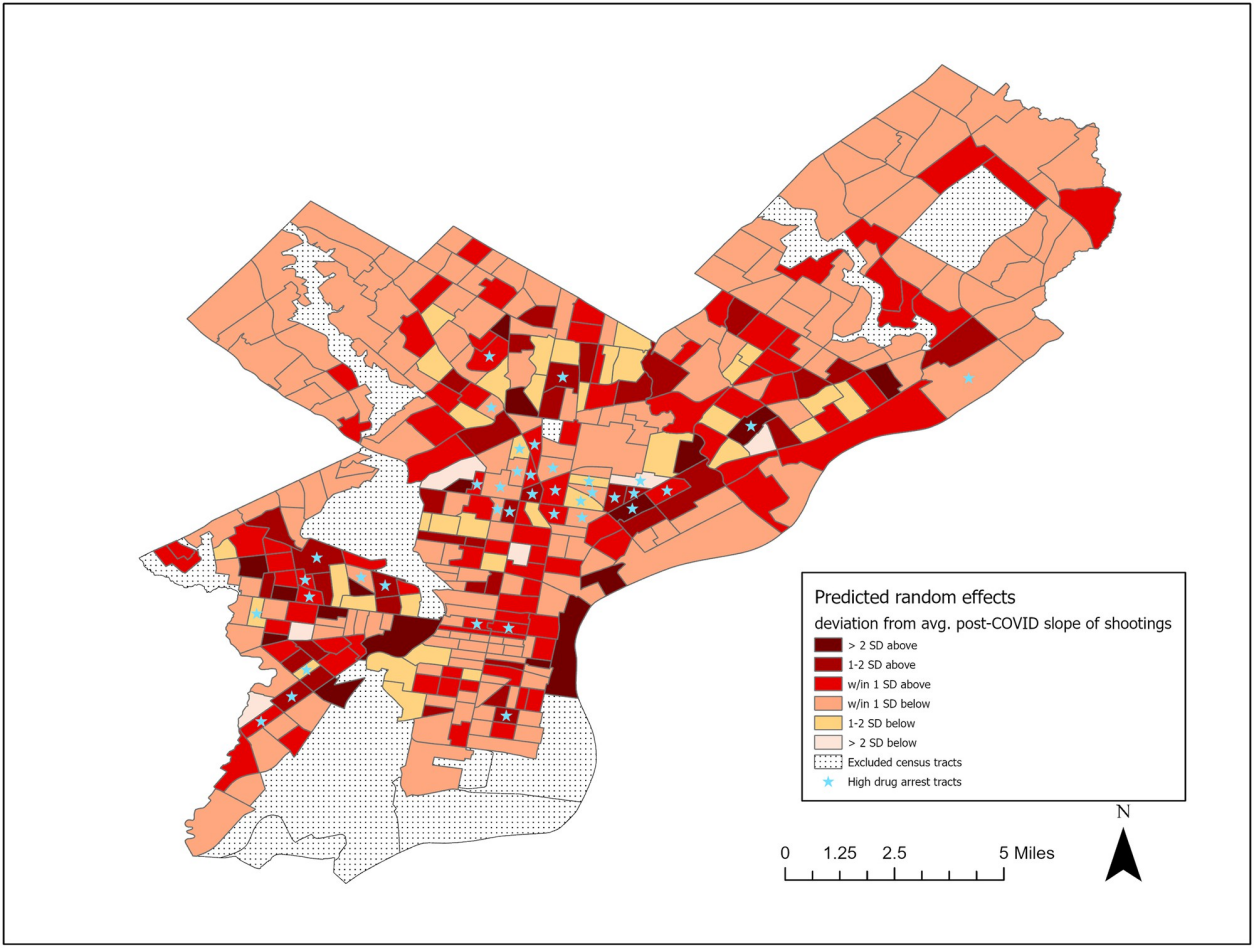
Model three predicted random effects. Census tract shapefile obtained from U.S. Census Bureau.

Model Three included random effects for both the *timepre* and *timepost* slopes. The *timepost* slope variance was relatively larger than the variance of the *timepre* slope, indicating greater variability in neighborhood shooting pathways after the onset of COVID-19. Fig 2 maps the random effects for the *timepost* slope with stars to denote the high-drug arrest tracts for reference. The red shaded values indicate a neighborhood was higher than the average post-COVID slope of shootings, whereas the orange and tan shaded values indicated a neighborhood was below the average post-COVID slope. Moran’s I tests were used to assess potential residual spatial autocorrelation. Each period’s residuals were assessed, with four bimonthly periods exhibiting significant residual spatial autocorrelation, though these values were relatively small (ranging from I = -0.05 to I = -0.09, pseudo-p < .05).

### Research question 5—What community correlates best predict variability over time

**Fig 3:**
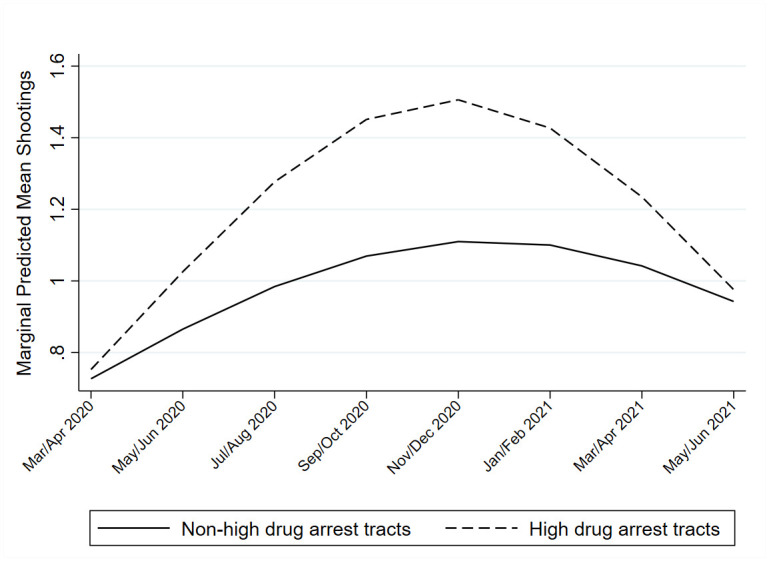
Predicted shootings post-COVID onset for high-drug arrest tracts and non-high drug arrest tracts.

Table 3 contains results for Models Four through Seven, which attempt to predict the variability in shooting slopes over time. Given the significant main effects of concentrated disadvantage and racial makeup, cross-level interactions with each of these variables and the time slopes were tested. Neither group of interaction terms were significant. However, the cross-level interaction of the drug market indicator with each time slope revealed that the effect of being a high-drug-arrest neighborhood on shootings increased with each passing period after, but not prior to, the onset of COVID-19. Furthermore, the negative coefficient of the drug market-*timepost*^*2*^ interaction suggests the high-drug arrest tracts that increased faster post-COVID onset also slowed faster toward the end of the time period. Coupled with the non-significant main effect of the drug market indicator, this suggests that while there was no difference in where drug market and non-drug market neighborhoods started out in 2020, their respective shooting rates evolved at different speeds as the pandemic unfolded. A plot of the adjusted marginal predictions of shooting rates for the significant cross-level interaction term with the post-COVID slope is included in Fig 3.

**Table 3:** Mixed effects models predicting shooting rates in Philadelphia neighborhoods, interactions with time.

Shootings	Model Four			Model Five			Model Six			Model Seven		
	*Coef*.	*SE*	*IRR*	*Coef*.	*SE*	*IRR*	*Coef*.	*SE*	*IRR*	*Coef*.	*SE*	*IRR*
Timepre	0.019***	.006	1.02	0.019***	.004	1.02	0.017***	.004	1.02	0.022***	.004	1.02
Timepost	0.163***	.048	1.18	0.120***	.036	1.22	0.235***	.034	1.27	0.245***	.038	1.28
Timepost^2^	-0.021**	.007	0.98	-0.024***	.006	0.98	-0.030***	.005	0.97	-0.033***	.006	0.97
PK Black X Pre	0.001	.007	1.00									
PK Black X Post	0.070	.061	1.07									
PK Black X Post^2^	-0.008	.009	0.99									
Concdis X Pre	-0.004	.004	0.99									
Concdis X Post	0.055	.036	1.06									
Concdis X Post^2^	-0.007	.005	0.99									
DM X Pre				-0.008	.009	0.99						
DM X Post				0.158*	.073	1.17						
DM X Post^2^				-0.023*	.011	0.98						
311 X Pre							-0.005	.004	0.99			
311 X Post							0.051	.050	1.05			
311 X Post^2^							-0.004	.008	0.99			
Stops X Pre										-0.007*	.003	0.99
Stops X Post										0.132*	.059	1.14
Stops X Post^2^										-0.024*	.010	0.98
Constant	-9.573***	.067		-9.578***	.061		-9.596***	.058		-9.566***	.060	
*Random effects*												
Variance(cons.)	0.249			0.246			0.243			0.245		
Variance(timepre)	0.0003			0.0003			0.0003			0.0004		
Variance(timepost)	0.003			0.003			0.003			0.003		
N obs.	9,620			9,620		9,620			9,620			
N groups	370			370			370			370		
*LL(df)*	-8748.1(24)			-8748.3(22)			-8748.3(22)			-8745.0(22)		
*AIC*	17544.2			17540.6			17540.6			17534.0		
*BIC*	17716.3			17698.4			17698.3			17691.8		

Notes.

* *p* < 0.05,

** *p* < 0.01,

*** *p* < 0.001.

All models control for covariates included in Models 2 and 3. Concdis refers to concentrated disadvantage. DM refers to drug market tracts.

**Fig 4:**
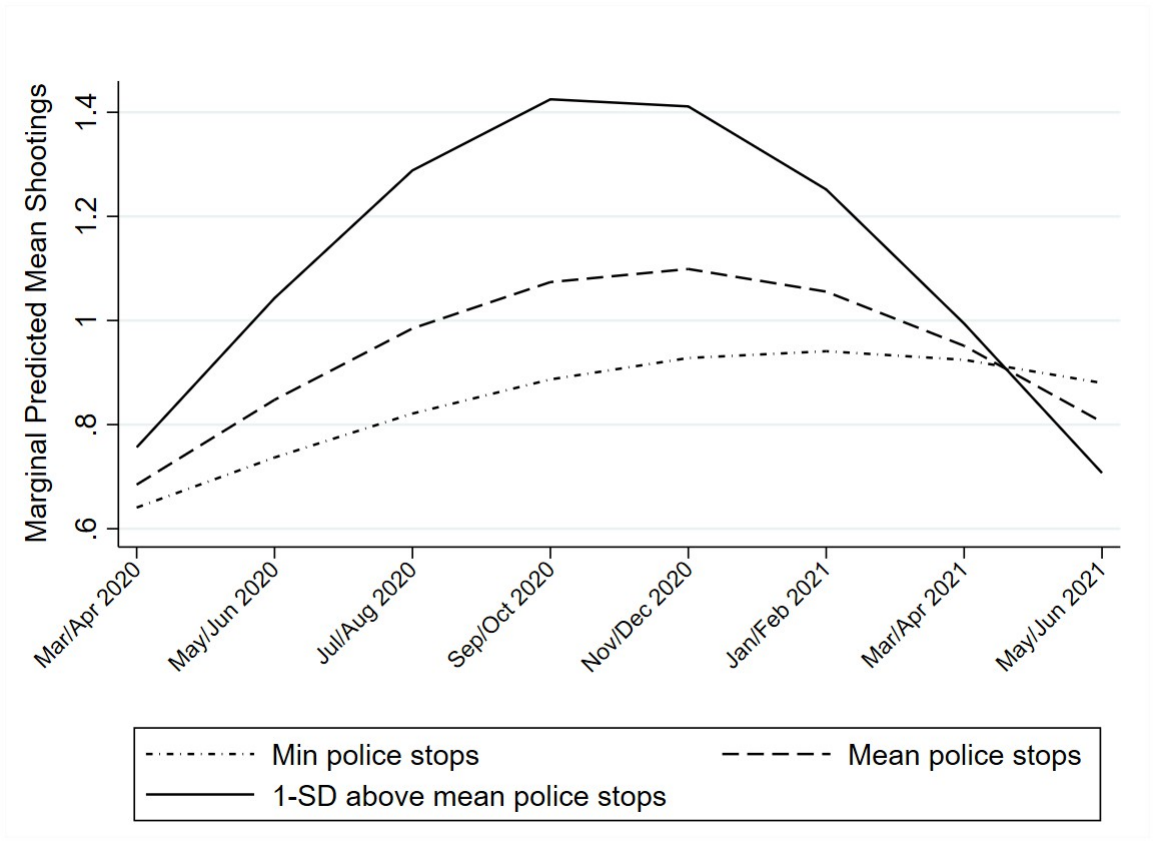
Predicted shootings post-COVID onset for varying levels of police stop activity. Min refers to tracts at the minimum level of police stops in the sample. SD refers to standard deviation.

Model Six included the cross-level interaction between physical incivilities and the time slopes, with no interaction term being significantly different from zero. The final model included interaction terms between the time-varying measure of police investigatory stops and all time slopes. All interaction terms were significant. For the post-COVID period specifically, the interaction with the timepost slope was positive, indicating that tracts with more stops increased in shootings at a faster rate than those with fewer stops. The negative *timepost*^*2*^ interaction coefficient further suggests that the tracts that increased at a faster rate also decreased faster toward the end of the study period. This trend is displayed graphically in Fig 4.

Model fit statistics, including the Bayesian Information Criteria (BIC), Akaike Information Criteria (AIC), and model log likelihood are included at the bottom of Tables 2 and 3. BIC values on their own are not interpretable, however, they allow for comparisons between models, with lower values indicating better model fit. The BIC for the unconditional growth model (Model One) was 18747, and dropped nearly 6% to 17668 for the full random intercepts only model without cross-level interactions. The model introducing random slopes had a slightly higher BIC (difference of 7) and slightly lower AIC (difference of 7) than the random intercepts-only model. The four models adding the individual cross-level interactions all had slightly higher BIC values than the random slopes model without the cross-level interactions (differences ranging from 16 to 41), suggesting that the models did not explain enough of the variation relative to the number of parameters being estimated. Multicollinearity was also assessed among the independent variables. All variance inflation factor (VIF) scores were less than five (mean 2.0, 1.2 (min)—4.6 (max)).

## Discussion

In this study, because we were interested in understanding how the COVID-19 pandemic influenced the structural, demographic and place-based factors typically associated with violence, we examined across-time and between-neighborhood differences in shootings in Philadelphia. Across the 4.5 year period, Philadelphia neighborhoods differed in both their initial levels of shootings immediately prior to the start of COVID-19, and in their rates of change in shootings as the pandemic progressed. The average rate of neighborhood shootings was markedly steeper after the onset of the pandemic, echoing what some research has found at the city-level [6]. Several factors related to neighborhood social structure and disorganization emerged as key predictors of these differences in shooting rates at the onset of the pandemic (i.e. March/April 2020). The extent of concentrated disadvantage and the racial makeup of a neighborhood predicted higher initial levels of shootings, but these effects did not change throughout the course of the pandemic. These findings correspond to what has been previously noted for cities like Indianapolis [42], and St. Louis [43], as well as cross-sectional analyses of Philadelphia [40]. Poor Black neighborhoods are at a much higher risk of gun violence than nonpoor, non-majority Black neighborhoods. Scholars seeking to understand the disparities in gun violence across communities have described plausible explanations for this pattern, including the social isolation of poor, urban communities that limits access to resources and positive role models [36, 37], or the cultural evolution of norms that are conducive to violence [38] or that weaken relationships with criminal justice agencies [39].

The detrimental impact of being embedded in a broader context of disadvantage and violence is also well-documented [73, 74], consistent with the finding that communities *surrounded* by more gun violence and more concentrated disadvantage were also at a higher risk for gun violence in the first two months of the pandemic. The significant effect of surrounding gun violence also echoes previous findings on the social contagion of violence, where focal census tracts in New York City were shown to be affected by levels of violence in the surrounding community (adjacent tracts) [71]. Relatedly, neighborhoods located further from drug markets experienced fewer shootings at the start of the pandemic. This further emphasizes the importance of the ecological context in which neighborhoods are situated. As shown in Fig 2, the tracts that were denoted as high drug arrest rate tracts tended to cluster in space, meaning that some areas of the city are likely to be more densely populated with illicit drug markets, buyers and sellers, and features of the environment that give rise to drug markets in the first place [61]. Drug markets may attract illicit drug activity that in turn gives rise to other types of criminal behavior [12]. While the broader community in which they are located also host residents with no ties to the illicit drug trade, some research suggests norms conducive to violence attributed to illicit drug trade can often bleed over into the surrounding community [64]. Neighborhoods situated in areas more reachable from all drug markets may be at increased risk of gun violence because they fall within the activity space of individuals or groups with ties to the drug trade, or because they are subject to a spillover of norms and behaviors conducive to violence.

Finally, the model results revealed that neighborhoods with higher police activity were associated with higher concurrent shooting activity at beginning of the pandemic. This is consistent with the idea that police resource allocation often follows the crime distribution across space. Indeed, this is the logic underlying hot spots policing [83], where areas that have relatively higher crime are targeted by relatively more policing activity. Given [20] finding that Philadelphia was one of a handful of cities driving an increase in the average weekly homicide rate (in a sample of 20 cities) from the end of May through June 2020, it would be reasonable to suspect that events surrounding the murder of George Floyd (i.e. changes in police activity and protest activity) may have corresponded to changes in shootings as well. In the current study, neighborhoods that had more police investigative stops in a bimonthly period had more shootings in the same period, and this relationship amplified over the course of the pandemic. This may suggest that violent neighborhoods that already had relatively higher levels of police attention at the start of the pandemic sustained even more as gun violence continued to rise. This is a potentially important finding, as some scholars have hypothesized that increases in violence resulted from *decreases in policing* due to police officers backing off proactive policing in minority neighborhoods (i.e. the Ferguson effect). However, as we reiterate in the limitations section, the width of the temporal units used in this study prevent us from exploring potential fine-scale changes in shootings that could have occurred in the immediate aftermath of George Floyd’s death.

An additional important finding is that neighborhoods varied in the relative acceleration of their shooting rates following the onset of the pandemic. In fact, there were greater differences between neighborhoods after the COVID-19 onset than before, meaning that the overall average increase in shootings after the start of COVID-19 could have been driven by relatively few exceptionally high-rate neighborhoods. Indeed, Fig 2 highlights how clusters of tracts located in North, Northwest, and Southwest Philadelphia exhibited higher rates of shootings than the “average” tract in Philadelphia during the pandemic. Of those measures tested, one of the only significant predictors of the differences in the evolution of shooting rates after COVID-19 was drug market status. This echoed Campedelli and colleagues’ finding in Chicago that neighborhoods experiencing reductions in assault were also likely to experience a significant reduction in drug crimes [7]. Interestingly enough, neighborhoods with active drug markets had a steeper increase in shootings during the pandemic than non-drug market neighborhoods, despite not being significantly different at the start of COVID (March/April 2020). This finding lends credence to claims by some scholars [e.g. 20] and law enforcement officials [84] that drug markets are driving part of the increase in shootings post-COVID onset.

A variety of potential explanations could undergird this finding. For one, the pandemic could have altered the perceived likelihood of punishment for drug organizations. The suspension of low-level, nonviolent arrests by the Philadelphia Police Department was widely publicized across news media outlets. The temporary suspension of nonviolent arrests could have emboldened some groups to become more active, thereby increasing their interactions with other sellers as they compete for the same buyers. It may be the general perception by drug organizations and new or potential sellers of a decrease in police activity (regardless of actual levels) were motivating criminal behavior. Widespread job loss and the economic downturn in the licit economy may have pushed some individuals into the illicit narcotics trade, thus further saturating the drug markets and also creating competition that resulted in violence. Additionally, economic strains might similarly have reduced the number of buyers, further increasing competition among sellers. With fewer potential guardians on the street, and place managers at local businesses curbing the drug trade, the violence potential of some drug gangs could have been exacerbated absent witnesses and other deterrents [11, 46]. Unfortunately, the necessary data (e.g., shooting motives, drug and gang-involvement in shootings, etc.) are currently lacking to examine any of the potential explanations outlined above.

Results from this study hold implications for two types of policy. The main effects of the models suggest certain types of *general* policies that may be geared towards reducing the disparate risk of gun violence across neighborhoods, while the cross-level interaction including the *timepost* variable suggests policies more focused on alleviating COVID-related dynamics of gun violence in certain communities.

The main effects of structural disadvantage and surrounding context suggest that gun violence prevention programs in general might be targeted towards neighborhoods most deeply entrenched in poverty and other structural burdens. Of course, high-poverty neighborhoods face myriad challenges regardless of their spatial location. Research has linked poor urban neighborhoods to limited access to key institutions, including supermarkets [85], green spaces [86] and retail employment opportunities [87]. At the neighborhood level, limited access to these types of prosocial institutions can inhibit collective health and wellbeing, and ultimately promote crime. Indeed, some public health researchers have posited that communities that are characterized by such economic depression do not possess the bandwidth (i.e., material and psychosocial resources) required to cope with environmental stressors [88].

It is also clear from the results of this study that spatial context matters. Scholars have long acknowledged that neighborhoods do not exist in isolation, but are rather interdependent units that affect, and are affected by, each other [89]. For communities geographically proximate to wealthy neighborhoods, this means extra-local opportunities and resources from which to draw on, and sustained exposure to prosocial role models [36]. Contrarily, communities that are embedded within regions of poverty are not only at an increased risk of violence based on the factors outlined above, but their local disadvantage is compounded due to the deprivation in surrounding neighborhoods as well. Although not shown, 57% of the high drug activity census tracts shown in Fig 2 as exhibiting above-average post-COVID rates of shootings also exceeded one standard deviation above the average level of concentrated disadvantaged in Philadelphia (compared to 49% of *non*-high drug activity tracts with above-average post-COVID shooting rates). These drug market census tracts also tended to be surrounded by other high-disadvantage tracts. Barring significant extra-local ties to organizations and city government, embedded neighborhoods are left to draw on their own limited resources and support for problem-solving [74]. And, absent long-term exposure to prosocial models of behavior, socially and culturally isolated communities may develop and transmit norms that are conducive to violence, such as gun-carrying behaviors, or using crime as a form of self-help [36, 90].

Policymakers can leverage this information by targeting gun violence prevention programs and other services to neighborhoods that are in a type of brokerage position within the broader network of neighborhoods. Harnessing the spillover effects of gun violence and social interactions in general by targeting key neighborhoods for prevention may work to not only alleviate gun violence risk within focal neighborhoods, but potentially disrupt gun violence risk across larger regions of a city. Particularly, investing in communities that are cutoff from tangential sources of support could be beneficial in reducing gun violence. For instance, Velez, Lyons, and Boursaw [91] found that extra-local investment in communities by virtue of residential mortgage lending resulted in later reductions in violent crime at the neighborhood level. Further, research has found that targeted city funding for community projects in Seattle was negatively associated with later neighborhood violent crime, and importantly, the protective benefits of targeted funding *amplified* with the accumulation of funds [92]. Overall, the type of investment needed to make a sustainable dent in gun violence goes beyond criminal justice interventions to cross-system investments that provide palpable resources to distressed neighborhoods [35]. This is suggestive of a public health framework that is comprehensive at its core, addressing multiple issues simultaneously for long-term change.

Future research will need to expand on the measures used in the current study to further understand the mechanisms connecting a community’s level of concentrated disadvantage to its own level of shooting rates. Qualitative inquiry into these processes could inform how best to disrupt pathways that promote higher rates of gun violence. Measures of neighborhood cultural frames, social capital and social cohesion might clarify some of the main effects of disadvantage highlighted here. While this study demonstrated the relevance of concentrated disadvantage on levels of gun violence in communities, other measures, such as levels of income inequality or segregation, might also be included to test alternative explanations to changes in shooting rates [7]. The covariates tested in this study were informed primarily by theories of social disorganization, incivilities, routine activities, and environmental criminology, though they are by no means exhaustive. Other theoretically-informed predictors of neighborhood gun violence should be included in future work, including more extensive measures of the theoretical concepts tested here. For example, additional measures capturing the routine activities of places, including receipt data to capture local business activity, employment data and working status of neighborhood residents, and objective measures of resident spatiotemporal mobility could be included in future research to tease out the complex relationship between concentrated disadvantage, COVID-19, motivated offenders, suitable targets and gun violence. The latter measures specifically could help gauge neighborhood levels of strain [93] or guardianship [11] that may lead to higher levels of violence. Additional measures related to environmental criminology, including criminogenic features of places and crime attractors and generators could also be included to provide more insight into important correlates of gun violence. Furthermore, research into the motivations of individual shooting incidents including the intersection with drug markets, will prove invaluable for not only understanding the relevant features of neighborhoods that play into rates of different types of gun violence, but also for developing more effective policies. Attention should also be given to how we might adapt existing models of gun violence prevention programs for unique times such as these, when many institutions that facilitate these programs (e.g. hospitals) are affected as well [94].

The significant cross-level interaction effect implies additional areas of focus for gun violence interventions during an exogenous shock like COVID-19. Taken together with Campedelli and colleagues’ [7] finding that Chicago communities experiencing a significant reduction in assault were also likely to experience a significant reduction in narcotics crimes, the increasing risk of being a neighborhood with an active drug market during the pandemic suggests that efforts related to disrupting drug organizations, or otherwise curbing violence stemming from drug markets, may go a long way towards quelling citywide increases in gun violence. Drug market risk of violence likely goes hand in hand with the structural factors that set the stage for the attraction and maintenance of illicit markets. Many, but not all, drug market tracts were located around the north central Philadelphia neighborhood of Kensington, a stark example of the agglomeration economy (i.e., mass collection of markets) of the illicit drug trade [95]. Fig 2 shows the geographic distribution of these high-drug activity tracts. This agglomeration factor could have promoted more violence than if the drug market landscape were more disparate. Indeed, the higher risk of neighborhoods located closer to all drug markets further emphasizes this point. The pandemic complicated narcotics policing because of the desire to mitigate the spread of COVID-19 [96]. Law enforcement and community partners may have to think creatively about ways to address issues related to drug markets during a pandemic. Ultimately, these models can only speak to correlations among neighborhood characteristics, rather than the mechanisms linking characteristics to initial levels and evolution of shooting rates over time. However, a clear implication from this study is that city officials should bolster the communities that appear to be particularly susceptible to detrimental outcomes following an exogenous shock such as COVID-19.

Of course, this study is not without limitations. First, the relative rarity of shooting events in Philadelphia precluded the use of a smaller temporal unit. By aggregating shootings to bimonthly units, rather than months or even weeks, any potential underlying variability is missed. The size of the temporal unit in turn prevented a more fine-grained examination of potential gun violence changes following the murder of George Floyd and subsequent protests. As detailed above, this occurred at the end of May 2020, meaning multiple factors connected to this tragedy, like protest activity or community-wide discontent towards law enforcement, could have theoretically affected the shooting rate. We lacked measures of these potential explanations, however, and could not test them directly. Additionally, our measure of neighborhood physical incivilities was captured by perceived issues raised by citizens to the local 311 hotline, rather than observed indicators of incivilities and disorder. It could be that actual disorder is more salient in explaining high levels of neighborhood gun violence. The current research is limited to a single city, and therefore may not be generalizable to other places. Philadelphia’s entrenched drug market landscape, including the neighborhood of Kensington, may further distinguish the city from other large urban areas. Finally, the method employed here does not allow for explanations of the causal impact of COVID-19 on shootings, rather, the analyses produced estimated trends, while testing correlates of those trends. Despite these limitations, this study adds value to the current body of work on COVID-19 and crime trends.

## Conclusion

This study explored the relative impact of several community features on rates of gun violence across neighborhoods and over time in Philadelphia during the COVID-19 pandemic. The results demonstrated here provide insight into several relevant aspects of communities that predict differences and growth in shootings, while also highlighting the need for inquiry into additional mechanisms and features of places. Gun violence dynamics likely played out in complex and heterogeneous ways across Philadelphia neighborhoods during the pandemic [7] and this study is a first step toward disentangling this complexity.

At the time of this writing, Philadelphia is still experiencing the effects of the pandemic. Neighborhood gun violence trends are slowing down from the dramatic increases experienced throughout 2020, yet levels remain higher than before the pandemic. In Philadelphia, it is clear that the rise in gun violence is unacceptable to city residents, but the resolution is less clear. We can glean from this study that a combination of efforts is required to address both the overall disparities in neighborhood risk of gun violence, and the rapid increases brought on by COVID-19. While long-term investment strategies should be deployed to address the former, short-term supports and efforts to combat violence associated with the drug trade may in turn alleviate the growth in gun violence in the wake of the pandemic.

## Supporting information

S1 Fig Model-implied average slope of shootings, 2017—June 2021. (TIF)

S1 Table Data sources. (DOCX)
