# Supplementary figures and images for: Community correlates of change: A mixed-effects assessment of shooting dynamics during COVID-19
Source: PLoS One. 2022 Feb 23;17(2):e0263777. doi: 10.1371/journal.pone.0263777 (PMC8865680; doi:10.1371/journal.pone.0263777)

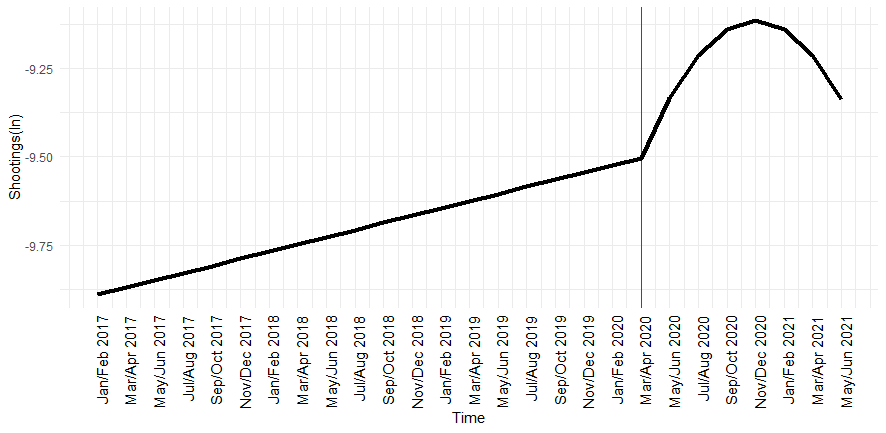

Supplement: S1 Fig — (TIF) [file pone.0263777.s001.tif]
