# Supplementary material for: Community correlates of change: A mixed-effects assessment of shooting dynamics during COVID-19
Source: PLoS One. 2022 Feb 23;17(2):e0263777. doi: 10.1371/journal.pone.0263777 (PMC8865680; doi:10.1371/journal.pone.0263777)
Supplement: S1 Table — (DOCX) [file pone.0263777.s002.docx]

# S2 Table. Data Sources

| **Data element/Variable** | **Data Source** |
| --- | --- |
| 2010 Philadelphia Census Tract boundaries | <https://www.opendataphilly.org/dataset/census-tracts>; https://www.census.gov/geographies/mapping-files/time-series/geo/carto-boundary-file.html |
| Shootings | https://www.opendataphilly.org/dataset/shooting-victims |
| Census measures (concentrated disadvantage, % renters, foreign born index, Peterson-Krivo black indicator) | https://data.census.gov/cedsci/ |
| Drug arrests | https://data.philadao.com/download.html |
| 311 calls for service | https://www.opendataphilly.org/dataset/311-service-and-information-requests |
| Police investigations | https://www.opendataphilly.org/dataset/vehicle-pedestrian-investigations |
